# Basic features of cellular inositol metabolism as revealed by a newly developed LC-MS method

**DOI:** 10.1042/BCJ20253028

**Published:** 2025-05-26

**Authors:** Xue Bessie Su, Valeria Fedeli, Guizhen Liu, Meike Marie Amma, Paraskevi Boulasiki, Jingyi Wang, Mariano Bizzarri, Henning Jacob Jessen, Dorothea Fiedler, Antonella Riccio, Adolfo Saiardi

**Affiliations:** 1 Laboratory for Molecular Cell Biology, University College London, London WC1E 6BTU.K.; 2 Institute of Organic Chemistry, Albert-Ludwigs-University of Freiburg, Albertstrasse 21, Freiburg 79104Germany; 3 Leibniz-Forschungsinstitut für Molekulare Pharmakologie (FMP), Robert-Rössle-Straße 10, Berlin 13125Germany; 4 Department of Experimental Medicine, “Sapienza” University of Rome, Systems Biology Group, Viale Regina Elena 324, Via A. Scarpa 14, Rome 00161Italy

**Keywords:** *chiro*-inositol, inositol depletion hypothesis, ISYNA1, Ino1, lithium, LC-MS, *myo*-inositol, Opi1, sugar, *scyllo*-inositol

## Abstract

Inositol plays key roles in many cellular processes. Several studies focussed on the quantitative analysis of phosphorylated forms of inositol, enabled by analytical tools developed to detect these highly charged molecules. Direct measurement of free inositol however has been challenging because the molecule is uncharged and polar. As a result, the mechanisms maintaining the homeostasis of the inositol remains poorly understood. In this study, we overcome these challenges by developing a quantitative liquid chromatography– mass spectrometry (LC-MS) protocol that can resolve and quantify the three main sugar molecules present inside cells: glucose, fructose and inositol, as well as distinguish the clinically relevant isomers of inositol: *myo-*, *scyllo-* and *chiro-*inositol. The quantitative power of the new method was validated by accurately monitoring the changes of inositol levels under well-established conditions in *Saccharomyces cerevisiae*, where the endogenous synthesis of inositol is increased in the transcription repressor *OPI1* knockout *opi1*Δ and decreased when wildtype yeast is fed with exogenous inositol. The method also revealed a new layer of regulation that takes place when exogenous inositol is added to further boost endogenous inositol synthesis in *opi1*Δ in a positive feedback loop. Analyses of mammalian cell lines provided many new insights into inositol metabolism. First, different cell lines displayed distinct sugar profiles and inositol concentrations and responded differently to inositol starvation. Second, mammalian cells can synthesise and import *scyllo-* but not *chiro-*inositol. Importantly, our method lent direct evidence to the previous hypothesis that lithium treatment could significantly reduce inositol levels in primary cortical neurons, thus diminishing the pool of free inositol available to the phosphoinositide cycle.

## Introduction

The cyclitol inositol (cyclohexane-1,2,3,4,5,6-hexol) is essential to eukaryotic cells in the form of the *myo*-inositol isomer (inositol thereafter) and provides the backbone for the synthesis of signalling molecules such as the water-soluble inositol phosphates (InsPs) [1] and the lipid-anchored phosphatidylinositol phosphates (PtdInsPs) [2]. InsPs and PtdInsPs regulate diverse signal transduction networks that control important cellular decisions. In the past few decades, extensive studies demonstrated that specific kinases and phosphatases carry out dynamic phosphorylation of InsPs and PtdInsPs, leading to a controlled flow of information within defined metabolic cycles [1,2]. Mechanisms regulating intracellular levels of the inositol scaffold, however, are less well understood, partly due to a common misconception that inositol levels are static in the cell and partly caused by a lack of reliable methods to quantify inositol levels from biological samples.

Intracellular levels of inositol are influenced by many pathways, including endogenous synthesis, recycling from dephosphorylation of InsPs and PtdInsPs, and transport in and out of the cells [3]. Classic as well as recent studies on the phosphatidylinositol cycle supported the idea that the availability of inositol directly controls the PtdInsP signalling [4,5]. The phosphatidylinositol cycle is initiated by the incorporation of free inositol in the lipids PtdInsPs, and the PtdIns(4,5)P_2_ that could be hydrolysed by phospholipase C (PLC) to release Ins(1,4,5)P_3_. Upon activation of calcium signalling, Ins(1,4,5)P_3_ is dephosphorylated by several phosphatases with the last step carried out by the inositol monophosphatase (IMPA or IMPase) to regenerate free inositol. Studies on the pharmacology and enzymology of the phosphatidylinositol cycle in the 1980 s led to the so-called ‘lithium inhibition hypothesis’, which proposed that the mood-stabilising effect of lithium (Li^+^) is based on its inhibition of IMPA activity and the resulting depletion of the available pool of inositol to regenerate PtdIns(4,5)P_2_ and Ins(1,4,5)P_3_ [5–7]. However, decades after the formulation of this hypothesis, direct evidence demonstrating the effects of Li^+^ on cellular inositol levels are still missing, hindering the development of new drugs to treat bipolar disorder (BD), which is currently estimated to affect 40 million people worldwide [8].

Another major lacuna in the understanding of inositol metabolism is a detailed analyses of the synthesis, transport and functions of other isomers of inositol. Different isomers of inositol are defined by the relative positions of the hydroxyl groups to the sugar ring. In the case of *myo*-inositol, the hydroxyl group on carbon 2 is axial to the plane of the ring, and the remaining five hydroxyl groups are in equatorial positions. A total of nine inositol stereoisomers could exist, some of which have been detected in eukaryotic cells. The *scyllo*- and D-*chiro*- isomers of inositol have emerged as potential pharmacological interventions to Alzheimer’s disease [9,10] and polycystic ovary syndrome (PCOS) [11–13], respectively, but little is known about the underlying mechanisms.

Since inositol is not UV-Vis active, detection of this molecule has relied on methods such as gas chromatography and chemical derivatisation, SAX-HPLC, enzymatic activity assays and bioassays based on the growths of inositol-auxotrophic yeast [14,15]. However, analytical chemistry-based assays suffer from major drawbacks such as laborious procedures and instrumental complexity. SAX-HPLC can only detect exogenous added radioactively labelled but not the endogenously synthesised inositol. Enzymatic and bioassays can be easily contaminated by other components in biological extracts. Recently, new chromatography matrices have been developed to facilitate quality control of sugar compositions and adulterations in soft drinks and milk formula. The capacity of these matrices to resolve inositol from other sugar molecules prompted a hydrophilic interaction liquid chromatography (HILIC) UHPLC-MS/MS approach to characterise the sugar and inositol isomeric compositions of human plasma [16]. We simplified this method and established a streamlined LC-MS protocol coupled with a reliable inositol cell extraction protocol to quantitatively measure intracellular inositol levels with high sensitivity and reproducibility. Using this method, we provide direct experimental evidence in support of several central concepts in the inositol field, from the presence of *scyllo*-inositol in mammalian cell extracts to experimental demonstration of the lithium inhibition hypothesis.

## Results

### Development of an LC-MS method for the detection of inositol

**Figure 1:**
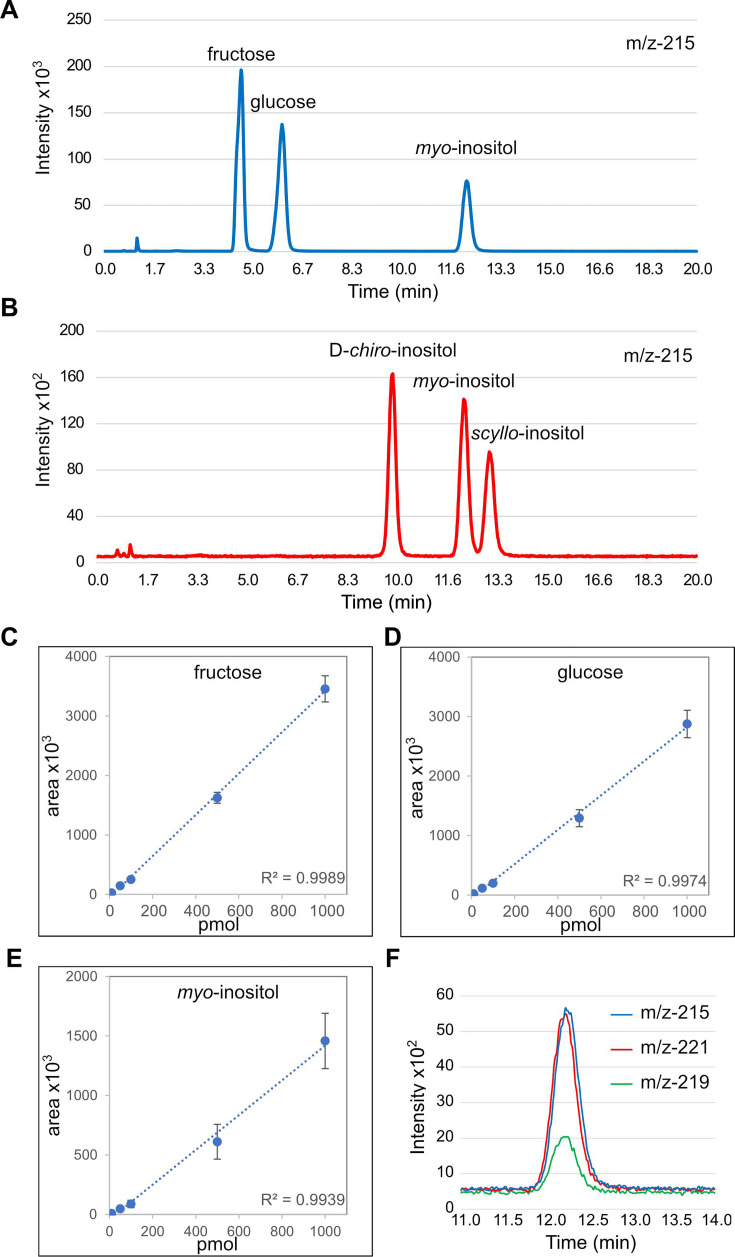
Validation of the LC-MS approach for the analysis of inositol. (**A**) Fructose, glucose and *myo*-inositol standards (1 nmol); and (**B**) *chiro*-inositol, *scyllo*-inositol and *myo*-inositol standard (0.1 nmol) were resolved on BEH Amide column. SIR chromatograms m/z of 215 [180+^35^Cl^−^] adducts are presented. Calibration curves for (**C**) fructose, (**D**) glucose and (**E**) *myo*-inositol standards showing the *R*^2^ values. (**F**) Representative analysis of co-injected myo-inositol (blue), ^13^C_6_-myo-inositol (red), ^13^C_2_-myo-inositol (green) standards (0.05 nmol of each standard). SIR chromatograms m/z of 215 [180+^35^Cl^−^], 221 [186+^35^Cl^−^] and 219 [182+^37^Cl^−^] adducts are presented. The intensity of the peaks are affected by the relative abundance of chloride isomers in nature (76% ^35^Cl^−^ versus 24% ^37^Cl^−^).

Our objective was to establish a sensitive and reliable LC-based mass spectrometry (MS) detection protocol for the sugar inositol, that relies on an inexpensive and user-friendly liquid chromatography instrument (LC-MS). We took advantage of the ability of chloride ions (Cl^−^) to form ionised sugar adducts, a process that greatly enhances their electrospray ionisation (ESI) sensitivity in the negative mode [17]. Using a Waters’s XBridge BEH Amide XP column and a mobile phase containing trace amount of guanidine hydrochloride (CH_5_N_3_·HCl), we successfully detected and resolved the three main sugar molecules present inside the cell: fructose, glucose and inositol (Figure 1A). Additionally, the protocol adequately resolved *chiro*-, *scyllo*- and *myo*-inositol isomers (Figure 1B). These results are comparable with those obtained from the complex and expensive UHPLC-MS/MS analyses [16].

We next tested the sensitivity and robustness of the LC-MS method by determining parameters such as calibration curve linearity, and lower and upper levels of quantitation (LLOQ and ULOQ). The LLOQ and ULOQ for all three sugar molecules were 10 picomole and 1000 picomole, respectively, and within this, range linear calibration curves were observed, with linear regression coefficients (*R*^2^) greater than 0.99 (Figure 1C, D and E and Table 1). Intra- and inter-day variations in retention times were below 10% using the same batch of column and mobile phase. Hence, our new LC-MS method allows a sensitive and robust quantification of fructose, glucose and inositol down to the picomole level.

**Table 1:** Validation of LC-MS as a quantitative method to measure fructose, glucose and myo-inositol.

Sugar	Linearity	Intra-day precision %		Inter-day precision %					
		Peak area	Retention time	Peak area			Retention time		
		Medium	Medium	Low	Medium	High	Low	Medium	High
Fructose	Y = 3474.9 x −52,526 (*R*² = 0.9989)	2.15	0.01	2.52	4.54	11.07	0.37	0.50	0.91
Glucose	Y = 2883.6 x −57,116 (*R*² = 0.9974)	2.71	0.03	24.04	28.43	13.87	0.37	0.36	0.83
*myo*-Inositol	Y = 1461.8 x −42,840 (*R*² = 0.9939)	3.27	0.04	53.97	59.78	27.63	0.37	0.41	0.83

Linearity and inter-day precision were determined for low (10 pmol), medium (100 pmol) and high (1000 pmol) concentration of each sugar molecule. Intra-day precision was measured for the medium concentration of each sugar molecule.

Chemical synthesis of non-radioactive isotopes of elements have been instrumental in the analyses of metabolites. Recently, a stable inositol isotopomer ^13^C_6_-inositol [18] was synthesised and applied to both nuclear magnetic resonance (^13^C-NMR) [19,20] and capillary electrophoresis (CE-MS) [21] studies to analyse inositol signalling and to determine the relative contributions of endogenously synthesised ^12^C-inositol and exogenously supplemented ^13^C_6_-inositol in InsPs metabolism [21]. We tested whether these isotopomers could be accurately resolved by our LC-MS system. As predicted by theoretical calculations, the chloride adduct of ^12^C-inositol was detected by a selected ion recording (SIR) measure of 215 (180 for inositol + 35 for ^35^Cl^−^), whereas the chloride adduct of ^13^C_6_-inositol was detected using an SIR of 221 (186 for ^13^C_6_-inositol + 35 for ^35^Cl^−^) (Figure 1F). Hence, ^13^C_6_-inositol can be added to growth media to quantify the amount of exogenous inositol entering the cell. We also considered the use of a second synthetic isotopemer, ^13^C_2_-inositol as a spike-in standard [22]. Although our protocol is highly reproducible, inter- and intra-day variations in instrumental performance such as ionising efficiency are inevitable and are best corrected by spiking the samples with an internal standard, which preferably is an isotopomer of the analyte of interest. Chloride has two stable isotopes in nature: ^35^Cl with a 76% abundance and ^37^Cl with a 24% abundance. Considering that an SIR of 217 would detect both the ^35^Cl^−^ adduct of ^13^C_2_-inositol and the ^37^Cl^−^ adduct of ^12^C_6_-inositol, we used an SIR of 219 to uniquely detect the ^37^Cl^−^ adduct of ^13^C_2_-inositol (Figure 1F). The spiking of the ^13^C_2_ internal standard hence allows for corrections of intra- and inter-day variations to improve the accuracy of quantification.

### Validation of inositol extraction protocol from cells

**Figure 2:**
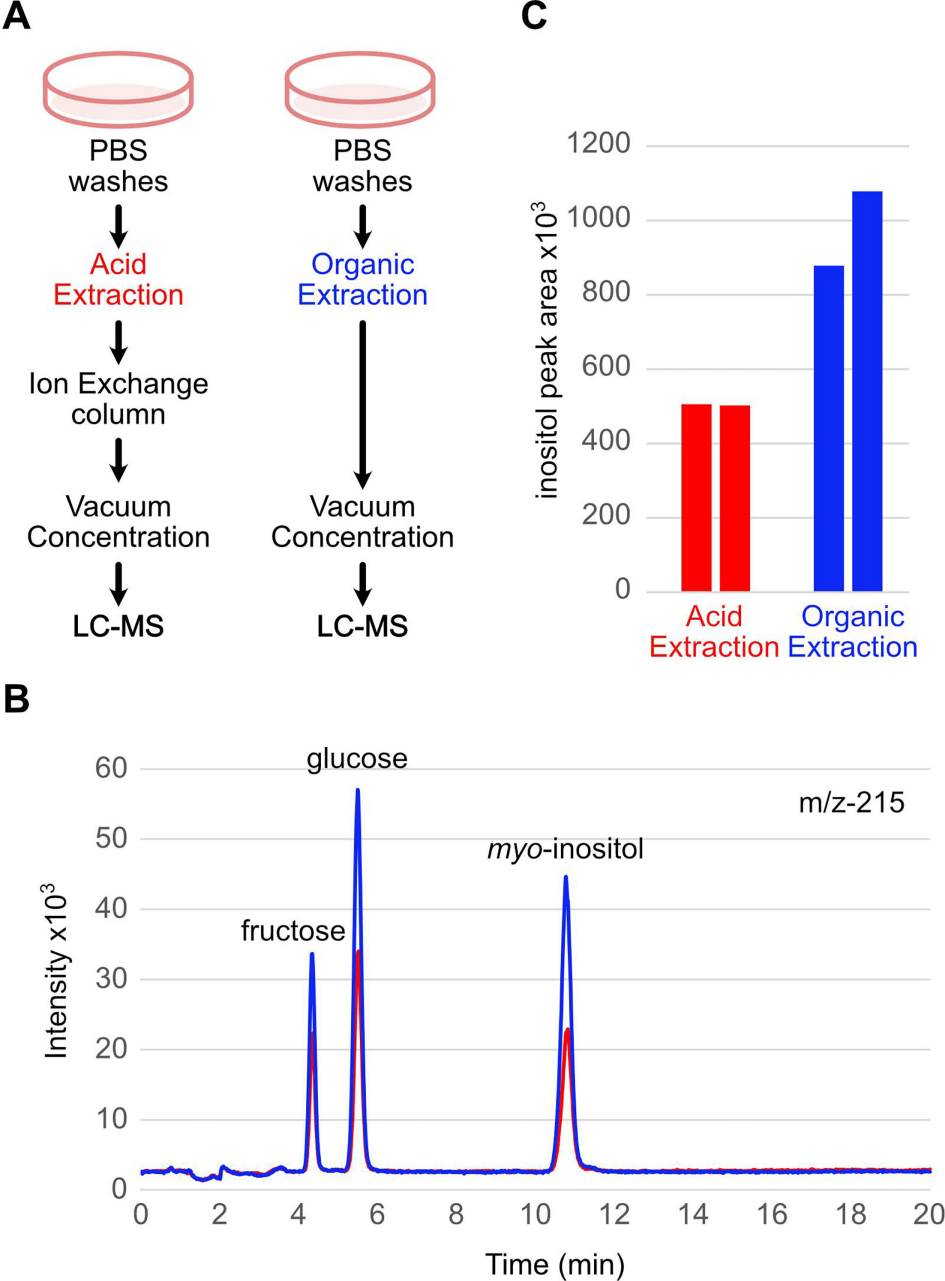
Validation of cellular sugar extraction methods. (**A**) Schematics describing the steps of acidic extraction (red) and organic solvent extraction (blue) methods. (**B**) Typical SIR chromatograms of m/z - 215 [180+^35^Cl^−^] adducts from acid extraction (red) and organic extraction (blue) are presented. (**C**) Peak areas of *myo*-inositol were determined from two independent extraction experiments in which acid extraction (red) and organic extraction (blue) were run in parallel using sister plates. SIR, selected ion recording.

We next sought to establish a reliable protocol to extract inositol from biological samples by adapting and comparing two existing workflows: acid extraction and organic extraction (Figure 2A). Since inositol is a polar molecule, it is routinely co-extracted with InsPs by strong acids such as perchloric acid [23]. Inositol binds poorly to anion exchange SAX-HPLC, leading to its early elution from the column after 2–3 min [23]. We performed perchloric acid extraction, with the added step of removing InsPs by gravity feed ion exchange resin [24] to avoid their detrimental effect on our column. In parallel, we performed an organic solvent extraction procedure that is frequently used by metabolomic studies aiming to quantify primary sugar and glycolytic intermediates such as glucose-6-phosphate and fructose-6-phosphate. The solvent we used contains acetonitrile, methanol and water in a 40:40:20 ratio, omitting formic acid from the original protocol [25,26]. Both acid and organic extraction protocols successfully recovered the three major sugars from HEK293T cells (Figure 2B andC). Since the organic extraction protocol routinely recovered 50–60% more inositol (Figure 2B and C) than the acid extraction protocol, we adopted the former for the rest of the study.

### Validating the technique using *S. cerevisiae*

**Figure 3:**
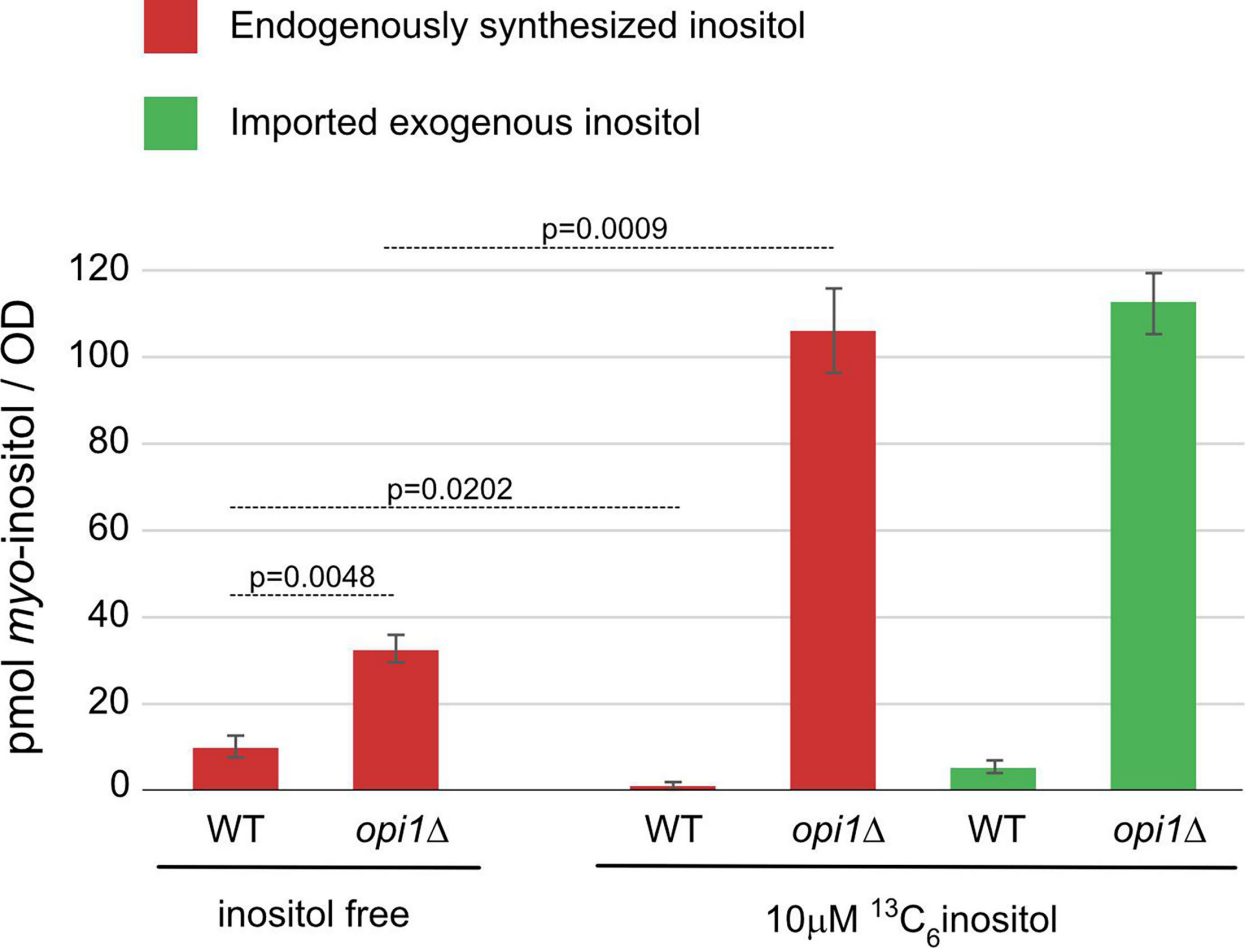
Analysis of inositol level in wildtype W303 and *opi1Δ::LEU2* yeast. Wild type W303 or *opi1Δ::LEU2* yeast cells were grown in complete synthetic media supplemented with or without 10 µM ^13^C_6_-*myo*-inositol. Sugar molecules were extracted from logarithmically growing yeast culture (OD_600_=20) and analysed by LC-MS. This analysis revealed that exogenously added ^13^C_6_-*myo*-inositol (green) represses endogenously synthesized *myo*-inositol (red) in wild type yeast and both *myo*-inositol import and synthesis are upregulated in *opi1Δ*. Data are represented as mean ± SEM from three biological repeats. P-values were determined by paired Student’s t-test.

Prior knowledge on cellular regulation of inositol levels was mostly gained from budding yeast *S. cerevisiae* [27]. *INO1* encodes the budding yeast homolog of inositol monophosphate synthase (named ISYNA1 in mammalian cells). *INO1* expression is tightly regulated at the transcriptional level. The addition of exogenous inositol represses its transcription in yeast. Opi1 is a well-established transcriptional repressor, and the *opi1Δ* yeast up-regulates the transcription of both *INO1* [28] and *ITR1*, which encodes the yeast inositol permease [29]. Bioassay and radiolabelled inositol measurements confirmed that endogenous synthesis and import of inositol are both up-regulated in *opi1Δ* yeast [29,30]. We used these well-established observations to validate our method. Wildtype and *opi1Δ* yeast cells were grown in inositol-free media or in media supplemented with 10 μM ^13^C_6_-inositol, and sugar extraction was performed by incubating yeast cells overnight in organic solvent. Consistent with previous studies, endogenously synthesised ^12^C-inositol (red) was significantly decreased in wildtype yeast when 10 μM ^13^C_6_-inositol was added to the media (Figure 3). Moreover, both endogenously synthesised ^12^C-inositol (red) and imported ^13^C-inositol (green) showed the expected increase in *opi1*Δ yeast (Figure 3). We also noted that *opi1*Δ yeast lacked the ability to repress endogenous inositol synthesis in the presence of exogenous inositol but instead elicited a positive feedback response (Figure 3). Overall, our method showed consistent results with previous literature as well as revealed a new function of Opi1 in repressing endogenous inositol synthesis in response to external inositol in budding yeast.

### Analysis of the inositol profile of mammalian cells

**Figure 4:**
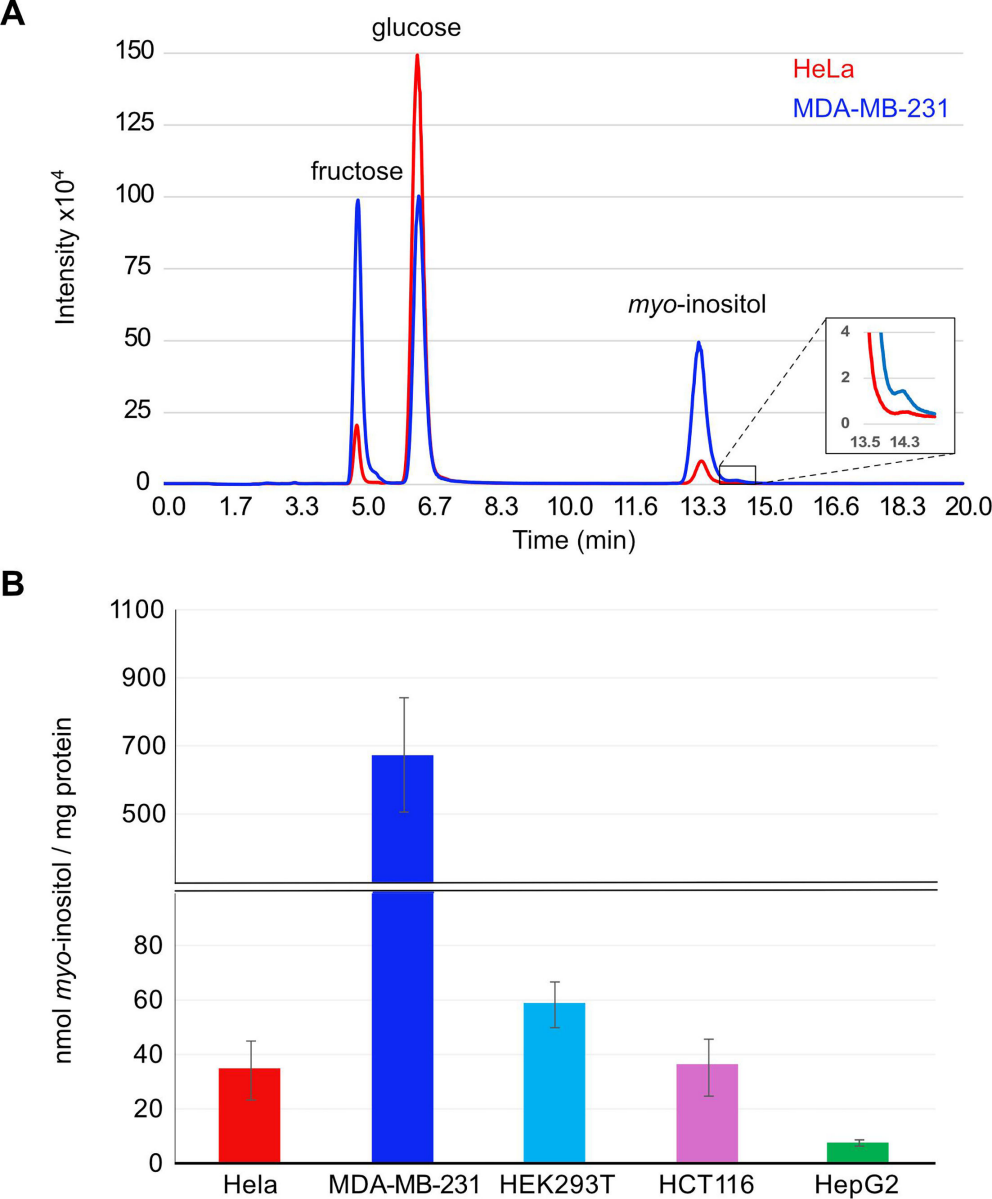
Analysis of inositol level in mammalian cell lines. Mammalian cell lines were maintained in standard culture conditions of DMEM supplemented with 10% fetal bovine serum. **(A)** Representative SIR chromatograms m/z of 215 [180+^35^Cl-] adducts of sugar extracts from a 10 cm plate of ~80% confluent Hela cells (red) and MDA-MB-231 (blue). The insert highlights a small bump that was observed at the expected elution time of *scyllo*-inositol. **(B)** The concentrations of endogenous myo-inositol extracted from five mammalian cell lines maintained using the same media and growth conditions. Data are represented as mean ± SEM from four biological repeats except for HepG2 in which three biological replicates were used.

Having validated our method in yeast, we extended the analysis to mammalian cells, in which much less is known about the basic regulatory mechanisms of inositol metabolism. We first examined whether inositol levels show variations in different cell lines, given that previous studies demonstrated that metabolism of glucose and fructose varies in a cell type-specific manner [31,32]. Five cell lines commonly used in research were studied, including HeLa (human cervical carcinoma), human MDA-MB-231 (breast adenocarcinoma), HEK293T (human embryonic kidney), HCT116 (human colon cancer) and HepG2 (human hepatocellular carcinoma). Cell lines were cultured under identical conditions of DMEM supplemented with 10% foetal bovine serum (FBS). The three main sugar molecules are well-resolved for all cell lines, as exemplified by the m/z-215 chromatograms of HeLa and MDA-MB-231 (Figure 4A). Nonetheless, both the relative abundance of these sugars and inositol levels varied widely between cell lines (Figure 4A and B). HepG2 showed the lowest amount of inositol at 7.7 ± 0.9 nmol/mg of protein (mean ± SEM, *n* = 3). HeLa, HEK293T and HCT116 had similar levels of inositol (34.9 ± 10.7 nmol/mg *n* = 5, 58.9 ± 8.3 nmol/mg *n* = 4 and 36.4 ± 9.9 nmol/mg *n* = 4, respectively). MDA-MB-231 had the highest level of intracellular inositol (672.6 ± 166.9 nmol/mg of protein *n* = 4). The almost 100-fold difference in intracellular inositol levels between HepG2 and MDA-MB-231 indicates that the homeostatic control of inositol metabolism and/or the kinetics of phosphoinositide and InsP cycles are likely to be very different in different cell types.

**Figure 5:**
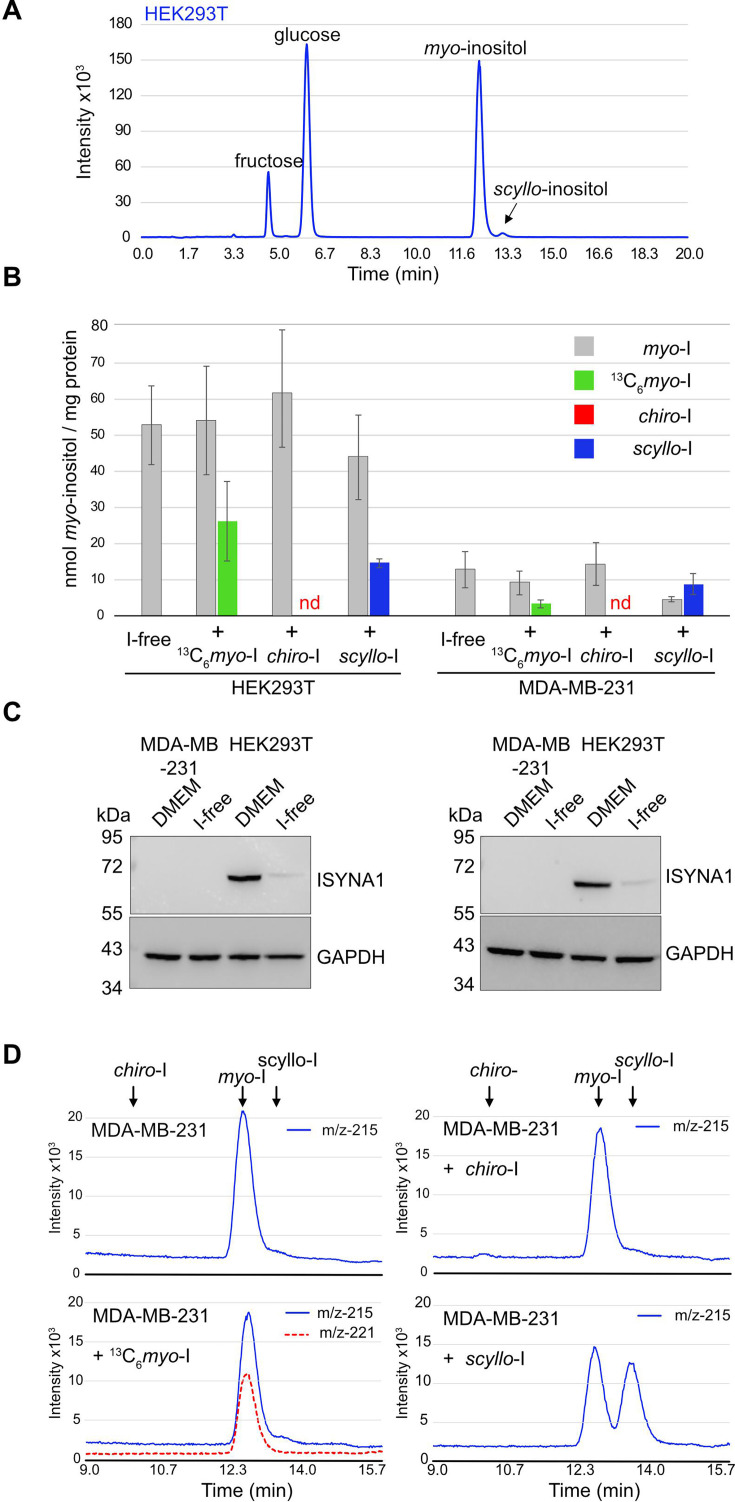
Presence of *scyllo*-inositol in HEK293T cell and cellular entry of both *myo*- and *scyllo*-inositol. (**A**) SIR chromatograms m/z of 215 [180+^35^Cl^−^] adducts of sugar extracts from two 10 cm plates of ~80% confluent HEK293T cells revealed a major peak of *myo*-inositol and a minor peak of *scyllo*-inositol. (**B**) Four parallel 10 cm plates of ~80% confluent HEK293T and MDA-MD-231 cells, were washed twice with PBS and shifted to inositol-free DMEM for 24 hours supplemented with 10 μM of ^13^C_6_-*myo*-inositol (^13^C_6_-*myo*-I), *scyllo*-inositol (*scyllo*-I), *chiro*-inositol (*chiro*-I), or the same volume of water (I-free). Sugars were then extracted and analysed. Reported are the concentration of endogenously synthesised myo-inositol (grey, *myo*-I), imported ^13^C_6_-*myo*-inositol (green) and imported *scyllo*-inositol (blue). D-*chiro*-inositol (red) did not enter either cell line (nd = not detectable). Data were represented as mean ± SEM from three biological repeats. (**C**) Anti-ISYNA1 western blotting was performed on cell extracts from HEK293T and MDA-MD-231 maintained in normal DMEM or in inositol-free DMEM (I-Free) for 24 h. Two independent experiments out of three are presented. GAPDH was used as loading control. (**D**) Representative SIR chromatograms m/z of 215 [180+^35^Cl^−^] adducts (blue) and m/z of 221 [186+^35^Cl^−^] adducts (red) of sugar extract from MDA-MB-231 untreated or treated for 24 h with 10 μM of ^13^C_6_-*myo*-inositol, *scyllo*-inositol and *chiro*-inositol. Arrows indicated the elution positions of *myo*-inositol (*myo*-), *scyllo*-inositol (*scyllo*-) and *chiro*-inositol (*chiro*-) standards. SIR, selected ion recording.

We next studied the endogenous synthesis of the *chiro*- and *scyllo*-inositol isomers, which are of clinical importance but poorly understood. We first noticed a small but reproducible peak (Figure 4A insert) eluting just after *myo*-inositol, which co-migrated with the *scyllo*-inositol standard (Figure 1B). The *scyllo*-inositol peak became more pronounced when sugar extracts were pooled from two ~80% confluent 10 cm plates of HEK293T cells (Figure 5A), and its level was ~3% of *myo*-inositol. In contrast, endogenous synthesis of *chiro*-inositol was not detected in any of the five cell lines analysed. Hence, *scyllo*- but not *chiro*-inositol appears to be commonly present in mammalian cells, albeit at a low concentration when compared with *myo*-inositol.

Another outstanding question in the inositol field is how cells respond to fluctuating inositol levels in the environment. To address this point, we performed an inositol starvation experiment by first growing cells in standard DMEM containing 40 μM inositol and then starving them in inositol-free DMEM media for 24 hours. Cell lines showed distinct responses to the starvation treatment. In HEK293T cells, intracellular inositol levels remained unchanged compared with non-starved cells, at 52.8 ± 10.9 nmol/mg protein (Figures 4B and 5B–I-free). MDA-MB-231 cells on the other hand, showed a 50-fold reduction in inositol levels to 12.8 + 5.0 nmol/mg protein (Figures 4B and 5B–I-free). The different responses to inositol starvation prompted us to examine ISYNA1 protein levels. Interestingly, ISYNA1 proteins were only detected in HEK293T but not MDA-MB-231 (Figure 5C, Supplementary FigureS1). Counterintuitively, shifting HEK293T for 24 hours in inositol free DMEM led to a reduction in ISYNA1 protein level. Hence, while HEK293T can respond to inositol starvation through endogenous synthesis, MDA-MB-231 lacks the ability to do so, thus explaining the differences in inositol starvation response.

We last examined the ability of cells to import different isomers of inositol from the culture media. To observe efficient import, we first starved cells in inositol-free DMEM for 24 hours and then added 10 μM myo-, *chiro*- or *scyllo*-inositol (Figure 5B and D). Of note, isotopic ^13^C_6_-*myo*-inositol was used because it would allow us to distinguish between imported and endogenously synthesised inositol (Figure 1F). Both HEK293T and MDA-MB-231 imported ^13^C_6_-*myo*-inositol efficiently, with HEK293T importing significantly more (26.2 ± 11.0 nmol/mg protein) than MDA-MB-231(3.3 ± 1.1 nmol/mg protein). Inositol import did not affect the level of ^12^C_6_-myo-inositol in either cell line, suggesting that unlike wildtype yeast, cells do not respond to exogenous inositol by diminishing endogenous synthesis. In contrast, *chiro*-inositol was not imported by either cell line (Figure 5B and D) nor did it affect *myo*-inositol levels. *scyllo*-inositol, on the other hand, promptly entered both cell lines, to a concentration similar as *myo*-inositol in HEK293T (14.3 ± 1.2 nmol/mg protein) and greater than *myo*-inositol in MDA-MB-231 (8.8 ± 2.9 nmol/mg protein). *Scyllo*-inositol also caused a mild although statistically insignificant reduction in *myo*-inositol levels in MDA-MB-231. In conclusion, our analyses of mammalian cells suggests that there is a high level of heterogeneity in inositol metabolisms in different cell lines. Moreover, although *chiro*-inositol showed therapeutic benefits in treating some human diseases, we did not observe its uptake in any of the cell lines we studied, suggesting that its therapeutic actions may be extracellular. The observations of *scyllo*-inositol synthesis and its prompt import by cells suggest that *scyllo*-inositol metabolism occurs in mammalian cells.

### Testing the inositol depletion hypothesis

**Figure 6:**
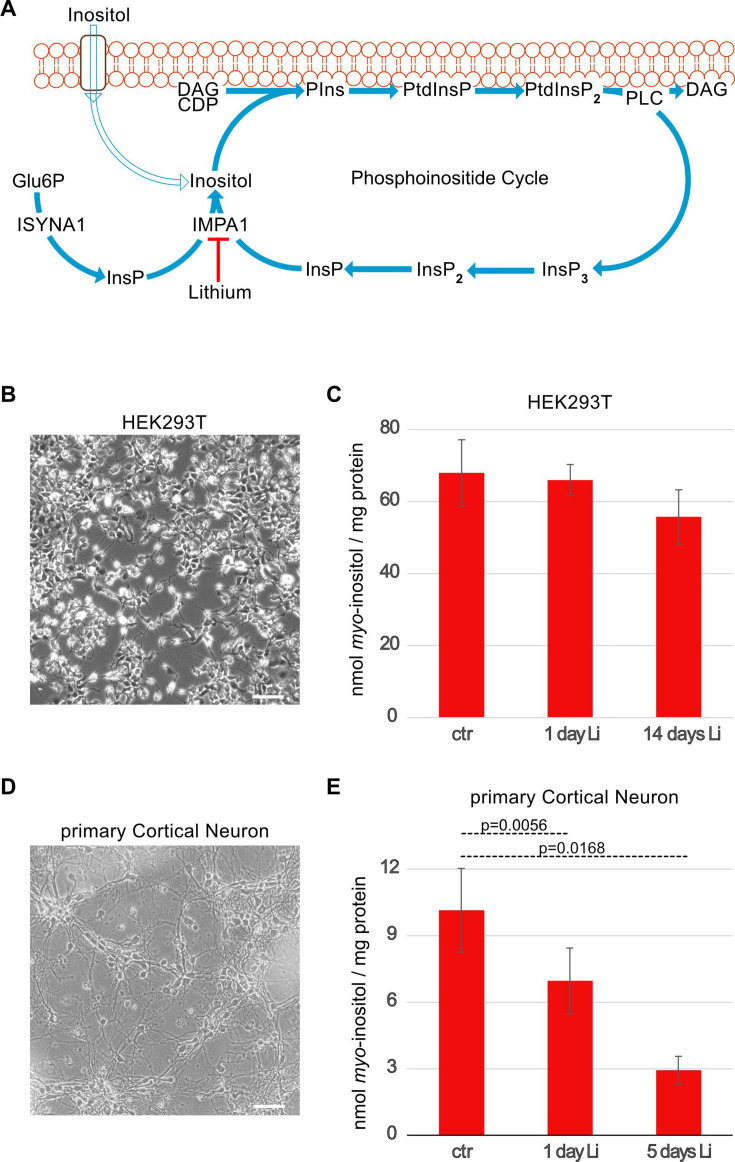
Experimental demonstration of the lithium depletion hypothesis: lithium treatment reduces inositol levels in primary cortical neuron. (**A**) This diagram illustrates the interconnected pathways of inositol import, endogenous synthesis and the phosphoinositide cycle, highlighting lithium’s point of action. Cells can acquire inositol from the extracellular environment (open arrow) via specific transporters [3]. In many cell types, inositol can also be synthesised endogenously by myo-inositol-3-phosphate synthase (MIPS or ISYNA1, Ino1 in yeast) from glucose-6-phosphate (Glu6P). Within the phosphoinositide cycle, phosphatidylinositol (PtdIns) is synthesised using inositol and cytidine diphosphate diacylglycerol (CDP-DAG, synthesised from phosphatidic acid), leading to the production of phosphatidylinositol phosphate (PtdInsP) and subsequently phosphatidylinositol-4,5-bisphosphate (PtdInsP₂). Phospholipase C (PLC) then cleaves PtdInsP₂, generating inositol-1,4,5-trisphosphate (InsP₃) and DAG. Inositol monophosphatase (IMPA1 in neurons) produces free inositol by dephosphorylating either inositol-3-phosphate, which is derived from MIPS activity, or inositol-1-phosphate, which originates from InsP₃ recycling through inositol-1,4-bisphosphate (InsP₂). Lithium inhibits IMPA1, thereby depleting the available inositol pool required for PtdIns synthesis, ultimately dampening the phosphoinositide cycle. (**B**) Microphotography of HEK293T cells, showing their characteristics epithelial morphology. (**C**) HEK293T maintained in normal DMEM were left untreated (ctr) or treated with 1 mM LiCl for 24 hours (1 day Li) and 14 days. (**D**) Microphotography of primary cortical neurons on day 11 post preparation, showing their electric maturity by presenting a fully developed network of dendrites and axons. (**E**) Primary cortical neurons maintained in normal MEM plus B-27 supplement at DIV 11 were either left untreated (ctr) or treated with 1 mM LiCl for 24 hours (1 day Li) or five days (5 days Li). Data are represented as mean ± SEM from three biological repeats. *P* values were calculated using paired Student’s t-test.

We last applied our method to test the long-standing inositol depletion hypothesis [6,33]. Lithium is known to effectively treat mood swings in BD patients. The inositol depletion hypothesis postulated that lithium acts as a non-competitive inhibitor of IMPA1 (Figure 6A), thus reducing the recycling of InsP back to inositol and ultimately diminishing the flux and thus the activities of the PtdInsP cycle [5–7,34]. To test the hypothesis, we initially treated HEK293T cells with the therapeutic dose of lithium (1 mM), either for 24 hours or over a prolonged period of 14 days, which is the established therapeutic duration for BD patients (Figure 6B) [35]. Intracellular inositol levels did not change significantly during this time (Figure 6C). We reasoned that the lack of effect may be due to differences in inositol metabolism in different cell types. Hence, we repeated the experiment in rat primary cortical neurons, which is a more appropriate disease model for neuronal disorders. Cortical neurons were plated and cultured for 10–11 days to achieve full differentiation characterised by a stable network of axons and extensive dendrite outgrowth (Figure 6D). Although a 24 hours treatment only diminished inositol levels mildly ~30% but significantly, a prolonged treatment (5 days), which is the maximum duration allowed for the primary cell cultures, caused a >70% drop in intracellular inositol levels (Figure 6E). Therefore, our LC-MS method showed directly that lithium reduces intracellular inositol levels, thus providing the long-waited experimental proof to Berridge’s proposed effect of lithium on the recycling of the inositol backbone from the phosphoinositide’s cycle. The observation of the cell-type specificity of lithium’s effect could be explained by differences in the rate of InsP turnover in HEK293T and cortical neurons, as the latter is known to have highly active phosphoinositide cycles, and thus, they are more likely to accumulate InsP when lithium acts as an uncompetitive inhibitor of IMPA1 [6,36].

## Discussion

Previously, intracellular levels of inositol were measured by techniques such as gas chromatography, SAX-HPLC, bioassays or enzymatic activity-based experiments. Although these methods have contributed greatly to our understanding of the basic mechanisms of inositol metabolism, they each suffer from major drawbacks. Gas chromatography and chemical derivatisation requires laborious procedures and thus lacks the potential to analyse large number of samples. ^3^H inositol-based SAX-HPLCs is unable to measure endogenously synthesised inositol. Bioassays and enzymatic assays are indirect and could be confounded by interfering compounds in the sample matrices. In this work, we developed a simple and straightforward LC-MS procedure that adequately resolve major sugars and inositol isomers. We also compared acid and organic extraction methods and established that a simple incubation of cells in a mixture of methanol:acetonitrile:water recovered satisfactory amount of sugar for LC-MS analyses.

The workflow accurately reported the expected changes in intracellular inositol levels in budding yeast. Furthermore, the use of non-radioactive isotopic inositol revealed that Opi1 not only regulates the expression of *INO1* and *ITR1* but also has a third function in repressing endogenous synthesis in response to external inositol. Future works combining yeast genetics and our LC-MS protocol will therefore shine lights on the new function of Opi1 and other unknown elements of inositol metabolism.

Our analyses of mammalian cell cultures revealed a high level of heterogeneity in inositol metabolism in different cell types, with intracellular inositol levels ranging from less than 10 to greater than 600 nmol/mg of proteins. This analysis should be expanded to additional cell lines in the future to gain a broad view of variations in basic inositol metabolisms in different cell types. Additionally, the analysis should be coupled with parallel examinations of the kinetics of PtdInsPs and InsPs metabolisms, to improve our understanding of how interconnecting cycles of inositol metabolic pathways co-ordinate to meet the physiological need of different cell types.

The heterogeneity in basic mechanisms regulating intracellular inositol was also demonstrated by the differences in how cells responded to inositol starvation. While HEK293T did not drastically reduce intracellular levels of inositol, MDA-MB-231 cells showed a 50-fold reduction. The ability of HEK293T to maintain inositol levels is most likely due to the expression of ISYNA1, which is notably absent from MDA-MB-231. Moreover, endogenous synthesis of inositol was unaffected by the addition of exogenous inositol in either cell line, suggesting that unlike yeast, ISYNA1 expression in mammalian cells is not controlled by extracellular levels of inositol. Interestingly, although MDA-MB-231 cells do not express ISYNA1, they have adapted to survive in both inositol-rich and -free media, tolerating dramatically different intracellular inositol levels. This raises the question of whether they operate an alternative enzyme/pathway of inositol synthesis, which a previous study had alluded to [21].

While *myo*-inositol is by far the most abundant inositol isomer present in mammalian cells, our LC-MS protocol revealed that *scyllo*-inositol is also commonly present in mammalian cells, albeit at a lower concentration compared with *myo*-inositol (<3% of the total pool of inositol). Furthermore, mammalian cells promptly import exogenous *scyllo*-inositol. These findings raise the possibility that *scyllo*-inositol might play important physiological functions in certain cell types or physiological contexts. These possibilities are worthy of further investigations because *scyllo*-inositol has been shown to effectively reduce the toxicity of amyloid β-peptide oligomeric aggregates found in Alzheimer’s disease [37]. In contrast, we did not observe either endogenous synthesis or import of *chiro*-inositol in the cell lines we examined. Although we cannot rule out that *chiro*-inositol metabolism could exist in other cell types, our findings point to a new possibility that *chiro*-inositol might exert its therapeutic action towards PCOS extracellularly [12,38], for example, by influencing the efficiency of sugars transporters, or by blocking the extracellular portion of receptors, or by inhibiting secreted enzymes/factors.

Pharmacological interventions such as lithium and valproic acid were shown to effectively manage BDs by dampening the kinetics of the phosphoinositide cycle. While the exact targets of valproic acid remain under debate [39–41], lithium is well-established to target the generation of inositol from InsP by uncompetitively inhibiting IMPA1 (Figure 6A) [42,43]. This unique uncompetitive inhibition increases in potency at higher InsP concentrations, suggesting that lithium’s effects are primarily confined to mature, actively firing neurons with high phosphoinositide turnover [44]. As a result, lithium selectively acts within the brain. In line with this, we did not observe a statistically significant reduction in inositol levels in the epithelial-like HEK293T cell line.

In previous experiments, we administered ³H-inositol to rat cortical neurons and measured inositol levels using SAX-HPLC. Contrary to expectations, lithium treatment did not lead to a detectable reduction in radioactive inositol levels [7]. This could be due to the fact that the technique only monitored the radiolabelled inositol taken up from the extracellular medium (Figure 6A) and was unable to detect the inositol synthesised endogenously. The contrasting results suggest that there are likely to be separate intracellular pools of inositol, only some of which control the substrate availability to the phosphoinositide cycle. For instance, it is possible that imported ³H-inositol resides within a transient pool that rapidly equilibrates with the extracellular environment through influx and efflux but is not efficiently transported to the subcellular compartments where phosphoinositide synthesis occurs. Further applications of our method, such as monitoring both endogenously synthesised and exogenously added ^13^C_6_-inositol, will therefore help to clarify the molecular mechanisms underlying other pharmacological interventions for BD patient such as that of valproic acid [45] or esbelen [46,47].

Decades of intense work have drawn many parallels between protein and inositol phosphorylation events, such as the reversible nature of this modification thanks to opposing activities of kinases and phosphatases, the existence of phosphorylation cascades and feedback loops, and their roles in regulating protein folding or enzymatic activities. However, most works to date have overlooked one potentially important parallel between the two events. While it is well-established that the intensity of a protein phosphorylation signal could be regulated at the most fundamental level by protein turnover, little consideration has been given to whether and how PtdInsPs and InsPs signalling might be modulated by fluctuations in inositol levels. As revealed by this study, intracellular inositol levels could be affected by endogenous synthesis, availability of exogenous inositol and recycling from InsP and PtdInsP cycles, in a cell-type specific manner. Hence, future works will be needed to carefully define the relative contributions of these different processes to inositol metabolism.

In conclusion, our newly developed workflow is low-cost, easy-to-implement and allows quantitative analysis of inositol in cell extracts. When combined with isotopic inositol, one can track both the endogenously synthesised and imported inositol, thus offering an unprecedented opportunity to study the interplay between the two sources of inositol. With the correct experimental design, one can apply the method to address many fundamental questions in inositol metabolism, such as how inositol levels are affected by internal cues and external stimuli, what roles they play in adjusting PtdInsP and InsP signalling pathways and whether there are different pools of inositol in different subcellular compartments to provide substrates for the demonstrated pools of PtdInsP and InsP [1,2]. The simplicity of the technique also means that it has a high-throughput potential, which will benefit both basic science studies and pharmaceutical research into this fundamental biological pathway.

## Materials and Methods

### Reagents and Materials

Ultrapure Milli-Q water [resistivity 18.2 MΩ·cm] was used to prepare all solution. The following chemicals were used: acetonitrile Optima LC/MS Grade, (Fisher Chemical, # 10055454), isopropanol Optima LC/MS Grade, (Fisher Chemical, #10001314), methanol Optima LC/MS Grade (Fisher Chemical, #10636545), diethylamine (Sigma-Aldrich, #471216), guanidine hydrochloride (Sigma-Aldrich, #G9277), *myo*-inositol (Sigma-Aldrich, #I5125), *scyllo*-inositol (Sigma-Aldrich, #I8132), D-*chiro*-inositol (Sigma-Aldrich, #74137), perchloric acid (Sigma-Aldrich, #244252), ethylenediaminetetraacetic acid (Sigma-Aldrich, #E8008), potassium carbonate (Sigma-Aldrich, #209619), lithium chloride (Sigma-Aldrich, #L9650). ^13^C_6_-Inositol and ^13^C_2_-inositol were synthesised as described previously [18,20].

### Cell lines, media and culture condition

HeLa (cervical carcinoma), HEK293T (embryonic kidney) and HCT116 (colon cancer) were acquired from (ATCC) [48], MDA-MB-231 (breast adenocarcinoma) was acquired from (ECACC) [49], and HepG2 (hepatocellular carcinoma) was a gift from a colleague [50], cells were grown and maintained in DMEM (Gibco, #21969035) supplemented with 10% foetal bovine serum (Gibco, #10500064) and 1% GlutaMAX (Gibco, #35050087) in a humidified cell culture incubator, under a 5% CO_2_ atmosphere, at 37°C. Inositol-free medium was prepared using inositol-free DMEM (MP Biomedicals, # 091642954) supplemented with 10% dialyzed FBS (Gibco, #26400044) and 1% GlutaMAX. Mycoplasma contamination was monthly monitored. Lithium treatment of HEK293T cells was conducted by adding 1 mM LiCl to the growing media for 24 hours. For chronic lithium treatment of 14 days, the cells were maintained in the constant presence of 1 mM LiCl throughout 2–3 cycles of cells splitting and culturing.

### Primary cortical neuron and lithium treatments

All animal studies were approved by the institutional animal care and use committees at University College London. All rat brain dissection were performed in authorised spaces at University College London (UK). Cortices were isolated from E17.5 Sprague Dawley rat embryos and maintained in a dissection buffer containing 2.5 mM Hepes (pH 7.4), 30 mM glucose, 1 mM CaCl2, 1 mM MgSO4, 4 mM NaHCO3 and 1× HBSS. The isolated cortices were enzymatically digested in a dissociation medium (1 mM Hepes pH 7.4, 20 mM glucose, 98 mM Na_2_SO_4_, 30 mM K2SO4, 5.8 mM MgCl2, 0.25 mM CaCl2, 0.001% Phenol red) containing 20 U/ml papain (Worthington Biochemicals, # LS003127) at 37°C for 25 minutes. Following digestion, the cortices were washed, mechanically dissociated and plated onto Nunc dishes (Thermo Fisher Scientific, #150460) pre-coated with 40 mg/ml poly-D-lysine (Sigma-Aldrich, #P7886) and 2 mg/ml Laminin (Corning, #354232). The cells were seeded in MEM (Gibco, #11095080) supplemented with 10% foetal bovine serum, 5% horse serum (Gibco, # 26050070), 1 mM glutamine and 1× penicillin-streptomycin. After 2–6 hours, the medium was replaced with Neurobasal medium containing (Gibco, #21103049) supplemented with 1 x B27 (Gibco, #17504044), 1 mM glutamine (Gibco, #25030149), 1× penicillin–streptomycin (Gibco, #15140148) and 10 mM FdU (Sigma-Aldrich, **#** 343333). Cultures maintained at 37°C in a 5% CO2 atmosphere. When the axon outgrowth was stable, which typically occurred on day 11–12, neurons were treated with 1 mM LiCl for 24 h or 5 days.

### LC separation and chromatographic mobile phase

Chromatographic separation was performed using an Alliance-HPLC system (Waters) using a XBridge BEH Amide XP 2.5 μm, 3.0 × 150 mm column (Waters, #186004869). The isocratic separation of sugars was achieved using a mobile phase consisting of 80:15:5 acetonitrile–water–isopropanol, 0.05% diethylamine and 1 μM guanidine hydrochloride, at a flow rate of 1 ml/min. The column temperature was maintained at 60°C. Mass spectrometry analysis was performed using an Acuity QDa (Performance) single quadrupole mass spectrometer (Waters). The ESI ion source was operated in the negative ion mode with cone voltage of 5.0 V and probe temperature of 600°C. Selected single ion recording (SIR) of 215 (180 for sugars + 35 for ^35^Cl^−^), 221 (186 for ^13^C_6_-inositol + 35 for ^35^Cl^−^) and 219 (182 for ^13^C_2_-inositol + 37 for ^37^Cl^−^) were used to record sugar molecules (glucose, fructose, *myo*-inositol), ^13^C_6_-*myo*-inositol and ^13^C_2_-*myo*-inositol, respectively. Data were processed using the Empower software (Waters).

### Method validation

The HPLC/MS method was validated by determining the following parameters: LLOQ, ULOQ, linearity and precision. Fructose, glucose and *myo*-inositol standards were diluted in 50:50 acetonitrile: water (v/v), from LLOQ to ULOQ (from 10 picomole to 1000 picomole). Intra-day reproducibility was determined by injecting 100 pmol of each sugar three times on the same day. Inter-day reproducibility was measured by injecting the standards on three different days, and the mean values were plotted to determine regression coefficient (R^2^). Precision was calculated as relative standard deviation of peak area and retention time of intra- or inter-day repeats.

### Extraction of sugar from cells

Adherent cultures of mammalian cells were grown in DMEM supplemented with glutamax and 10% foetal bovine serum. Typically, a 100-mm dish was used for sample preparation. Sub-confluent cultures were washed twice with cold phosphate-buffered saline and subsequently incubated in cold 40:40:20 methanol:acetonitrile:water for 10 min on ice. Soluble materials were transferred to Eppendorf tubes and the solvent was evaporated in a temperature-controlled (~45°C) speed vacuum. Insoluble materials that remained attached to the plate were washed twice with cold PBS and solubilised in radioimmunoprecipitation assay (RIPA) buffer (ThermoFisher, #89901) for the determination of protein amount using a Bradford assay (BioRad, #5000201) following manufacturer’s instructions.

Sugar extracts were dissolved in 50 μl 50:50 acetonitrile:water and kept refrigerated for at least 10 min or until analyses. Samples were centrifuged at 13,000 rpm for 5 min and soluble fraction were transferred to glass vials. Typically, 10 μl sample was injected. Sugar concentrations were calculated by comparing with the sugar standards injected on the same day and normalised to the amount of proteins in each sample.

To compare the acidic extraction protocol to the organic extraction methods, sister plates were extracted in parallel. For acidic extraction, sub-confluent cultures were washed twice with cold phosphate-buffered saline and subsequently incubated for 10 min on ice with 0.5 ml 1 M Perchloric acid supplemented with 5 mM EDTA. After transferring the acidic extract to an Eppendorf tube, it was neutralised using ~0.2 ml 1M potassium carbonate. The clean neutralised extract was passed through 0.5 × 1 cm Dowex AGI-X8 resin (BioRad, #1401444). Unphosphorylated inositol did not bind to the resin and was collected as flow through. Resins were washed twice with 0.5 ml of Milli-Q water. The flow through and the washes were pooled and evaporated in a temperature-controlled speed vacuum.

### Yeast culture and sugar extraction

The wildtype W303 yeast used in this study was *MATa leu2-3,112 trp1-1 can1-100 ura3-1 ade2-1 his3-11,15*. *OPI1* null was generated by amplifying the *opi1Δ::LEU2* knockout cassette from plasmid pUG73 [51] using primers *opi1Δ*_forward: TTAAAGCGTGTGTATCAGGACAGTGTTTTTAACGAAGATACTAGTCATTGCAGCTGAAGCTTCGTACGC and *opi1Δ*_reverse: GTATAATATTATTACTGGTGGTAATGCATGAAAGACCTCAATCTGTCTCGGGCATAGGCCACTAGTGGATCTG and transforming the PCR product into wildtype W303 yeast, using standard homologous recombination and selection procedures. Yeast cells were cultured in yeast nitrogen base without amino acids or inositol (Formedium, #CYN3702) supplemented with complete supplement mixture (Formedium, #DCS0019) and 2% glucose (Sigma-Aldrich, #G8270). 20 OD cells were harvested in mid-log phase by centrifuging at 4,000 rpm for 1 min and washing twice with 20 ml cold water. Cells were resuspended in 2 ml 40:40:20 methanol:acetonitrile:water, transferred to Eppendorf tubes and incubated horizontally at −20°C overnight. The suspension was centrifuged at 13,000 rpm for 10 min at 4°C, and the soluble fraction was dried in a speed-vacuum and processed as described for mammalian cells.

### Western blot assay

Proteins were extracted from one 10 cm plates of HEK293T and MDA-MB-231 using RIPA Lysis Buffer (Millipore, # 20–188) supplemented with PhosSTOP™ (Roche, #4906845001) and cOmplete™, EDTA-free Protease Inhibitor Cocktail (Roche, #04693132001). The protein extracts were quantified using the DC™ Protein Assay kit (Bio-Rad, #5000122). Proteins (30 µg) were resolved by electrophoresis using 4-12% Bis-Tris polyacrylamide NuPAGE gels (Invitrogen, # NP0321BOX) and transferred to Immun-Blot PVDF Membrane, (BioRad, #1620177). Membranes were blocked in 5% non-fat milk in TBS-T (0.1% Tween 20) and incubated overnight at 4°C with ISYNA1 (C-9) primary antibody (Santa Cruz, #sc-271830). After three washes in TBS-T, membranes were incubated with a secondary anti-mouse IgG1 antibody (Invitrogen, # PA-74421) for 2 hour at 4°C. GAPDH primary antibody (Invitrogen, # MA5-15738) was incubated for 1 hour at room temperature and after three washes in TBS-T, membranes were incubated with a secondary anti-mouse IgG1 antibody (Invitrogen, # PA-74421) for 1 hour at R.T.. Detection was performed using the Clarity™ Western ECL substrate (Bio-Rad, #170–5060) and images were acquired with the Alliance Q9 imaging system.

### Statistical analysis

Statistical results are expressed as the mean ± SEM. Paired Student’s t-test for parametric data was performed using Graph Pad Prism 9 (Graph Pad Software, San Diego, USA). For each experiment, details of statistical analyses are described in the text or figure legends.

## Supplementary material

Online supplementary figure

## Data Availability

The findings of this study are supported by the data within the article. The raw data and materials used in this study are available on request to corresponding author.

## Abbreviations

ESI: electrospray ionisation
IMPA: inositol monophosphatase
InsPs: inositol phosphates
LC-MS: liquid chromatography - mass spectrometry
PCOS: polycystic ovary syndrome
PLC: phospholipase C
PtdInsPs: phosphatidylinositol phosphates
SIR: selected ion recording
