# Supplementary material for: Basic features of cellular inositol metabolism as revealed by a newly developed LC-MS method
Source: Biochem J. 2025 May 26;482(11):675–90. doi: 10.1042/BCJ20253028 (PMC12203950; doi:10.1042/BCJ20253028)
Supplement: Online supplementary figure [file BCJ-482-11-BCJ20253028-s001.docx]

**Supplementary material**

**Basic features of cellular inositol metabolism as revealed by a newly developed LC-MS method.**

Xue Bessie Su^1^, Valeria Fedeli^1^, Guizhen Liu^2^, Meike Marie Amma^3^, Paraskevi Boulasiki^1^, Jingyi Wang^1^, Mariano Bizzarri^4^, Henning Jacob Jessen^2^, Dorothea Fiedler^3^, Antonella Riccio^1^, Adolfo Saiardi^1^ ^*^

^1^ Laboratory for Molecular Cell Biology, University College London, London WC1E 6BT, UK.

^2^ Institute of Organic Chemistry, Albert-Ludwigs-University of Freiburg, Albertstrasse 21, 79104 Freiburg, Germany

^3^ Leibniz-Forschungsinstitut für Molekulare Pharmakologie (FMP), Robert-Rössle-Straße 10, 13125 Berlin, Germany

^4^ Department of Experimental Medicine, “Sapienza” University of Rome, Systems Biology Group, Viale Regina Elena 324, Via A. Scarpa 14, 00161 Rome, Italy

^*^ Corresponding:

Adolfo Saiardi Email: [a.saiardi@ucl.ac.uk](mailto:a.saiardi@ucl.ac.uk)

**
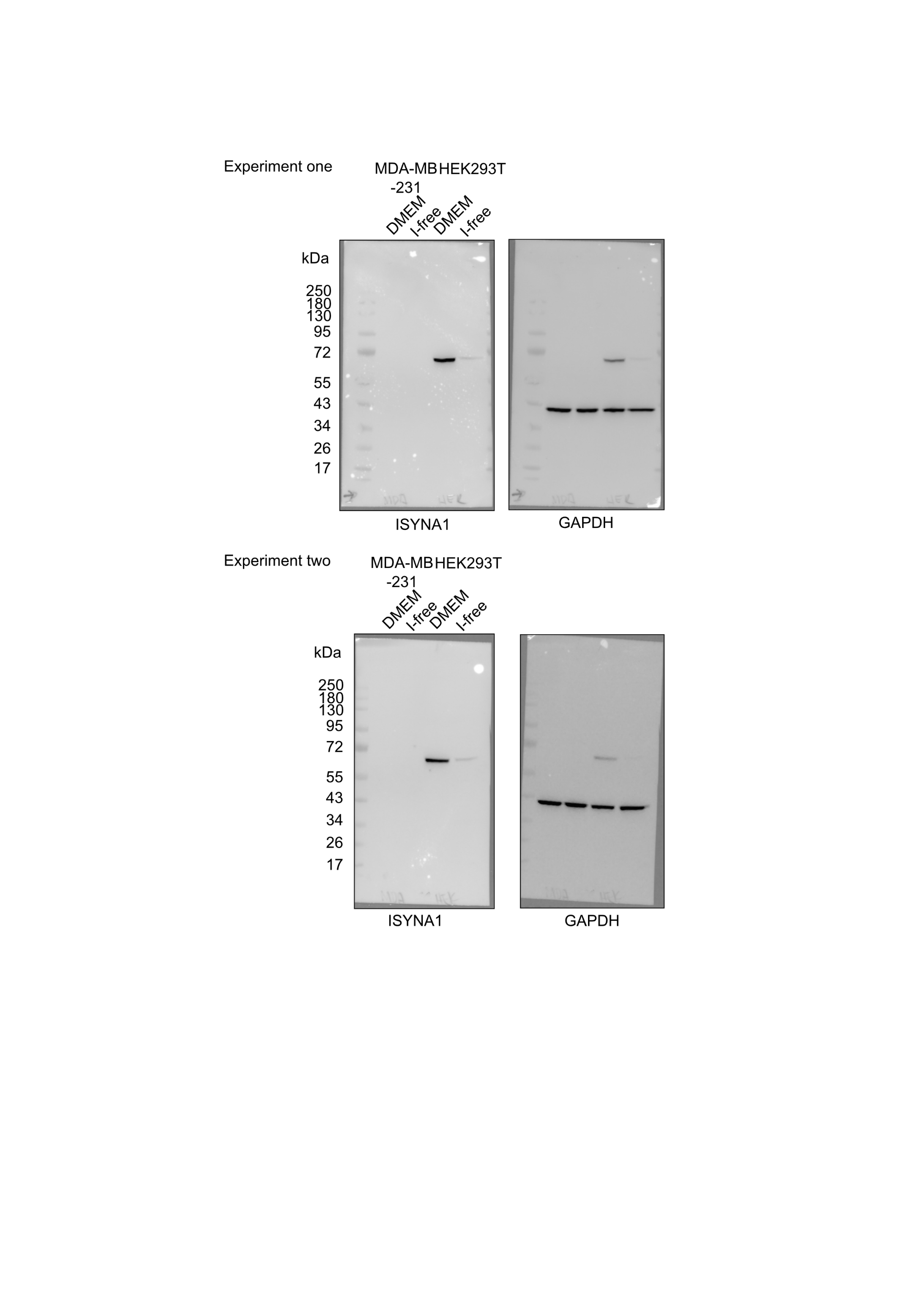
**

**Figure S1.** **Uncropped and unedited versions of the ISYNA1 and GAPDH Western blots**

First an anti-ISYNA1 antibody was used on cell extracts from HEK293T and MDA-MD-231 maintained in normal DMEM or in inositol-free DMEM (I-Free) for 24 h. Subsequently, an anti-GAPDH blotting was performed to verify equal loading. During the visualizing of the anti-GAPDH signal a residual detection of the previously used anti-ISYNA1 antibody could be see Uncropped and unedited versions of both experiments reported in figure 5C are presented.
